# The complete chloroplast genome of *Saussurella borneensis* (Orthoptera: Tetrigoidea) from China and its phylogenetic analysis

**DOI:** 10.1080/23802359.2021.1966336

**Published:** 2021-08-24

**Authors:** Wei-An Deng, Rong-Jiao Zhang, Xiao-Dong Li, Lei Xin

**Affiliations:** a Ministry of Education, Key Laboratory of Ecology of Rare and Endangered Species and Environmental Protection (Guangxi Normal University), Guilin, PR China; b School of Chemistry and Bioengineering, Hechi University, Yizhou, PR China; c Guangxi Key Laboratory of Rare and Endangered Animal Ecology, Guangxi Normal University, Guilin, PR China; d College of Life Sciences, Guangxi Normal University, Guilin, PR China

**Keywords:** Tetrigidae Batrachidinae, *Saussurella borneensis*, mitogenome, phylogenetic analysis

## Abstract

The mitochondrial genome (mitogenome) of *Saussurella borneensis* (Orthoptera: Tetrigoidea) was determined and analyzed. The complete mitochondrial genome is 16,006 bp in length, consisting of 37 genes, including 13 protein-coding (PCGs), 22 tRNA, 2 rRNA genes as well as an A + T-rich region. Ten PCGs initiated with a typical ATN codon (one with ATC, two with ATA, two with ATT, and five with ATG) and 13 terminated with complete stop codons. The overall nucleotide composition was 42.97% for A, 17.61% for C, 11.62% for G, and 27.8% for T. Phylogenetic analysis of *S. borneensis* fully resolved it in a basal branch sister to *Tripetaloceroides tonkinensis*. This data increase the bioinformatics of the Tetrigidae, and improves our understanding of the phylogenetic status of *S*. *borneensis* in the Tetrigoidea.

*Saussurella borneensis* Hancock, 1912, is classified in the subfamily Batrachidinae of Orthoptera. This subfamily currently contains 3 tribes and 25 known genera worldwide; the genus *Saussurella* Bolivar includes twelve species endemic to Southeast Asia (Deng 2016; Zha et al. 2020). However, there is little information on the systematic position of *S. borneensis* within the Tetrigidae (Zhang et al. 2020). To date, no mitochondrial sequence has been reported for the Batrachidinae (NCBI, last visited on May 2021). To further advance the evolutionary studies of the Batrachidinae, we sequenced, assembled and performed a phylogenetic analysis of the mitochondrial genome of *S. borneensis* (GenBank accession No. MZ169555) to contribute to the evolutionary systematics of the Batrachidinae.

The samples of *S. borneensis* were collected from Nonggang Nature Reserve in Guangxi province of China (22.474261°N, 106.957389°E) in May 2020 and the voucher specimen was deposited in Entomological Museum of Hechi University (EMHU) (the voucher No. bb1, Hechi University, Yizhou, China, Deng WA, dengweian5899@163.com). Total genomic DNA was extracted from the legs of an adult specimen of *S. borneensis* using the DNeasy Blood & Tissue Kit (Qiagen, Dusseldorf, Germany) according to the manufacturer’s instructions. The genomic DNA was sequenced using 150 bp PE on the Illumina Novaseq platform (Personalbio, Shanghai, China). The mitogenome was de novo assembled using A5-miseq v20150522 (Coil et al. 2015) and SPAdes version 3.9.0 (Bankevich et al. 2012), and all genes were annotated with the MITOS Web Server (Bernt et al. 2013).

The complete mitogenome of *S. borneensis* is 16,006 bp, and contains 13 protein-coding genes (PCGs), 22 tRNAs, 2 rRNA (*rrnS* and *rrnL*), and an A + T-rich region. The composition of the mitogenome is 42.97% A, 17.61% C, 11.62% G, and 27.8% T, showing an A + T bias (70.77%). Nine PCGs and fourteen tRNAs were transcribed from the majority strand, while the remaining four PCGs (*ND1*, *ND4*, *ND4L*, and *ND5*), eight tRNAs and two rRNAs were located on the minority strand. Ten PCGs initiated with a typical ATN codon (one with ATC, two with ATA, two with ATT and five with ATG), whereas the *ND1*, *ND4*, and *ND4L* genes started with TTG. Thirteen terminated with complete stop codons (one with AAC, AAG, ACA, ATG, ATT, GCA, GTA, TGA, and TGT, respectively, two with GAA and two with TTA). Twenty-two tRNA genes were found interspersed in the mitogenomes of *S. borneensis*, which ranged in size from 62 bp (*trnH*) to 70 bp (*trnK* and *trnV*). The two rRNA genes, i.e. *rrnS* (742 bp) and *rrnL* (1371 bp), were located between the *trnL1* and an A + T-rich region, and separated by the *trnV* gene. The gene composition, order and orientation of *S. borneensis* was the same as the mitogenomes of other tetrigid species, such as *Tripetaloceroides tonkinensis* (MW770353) and *Tetrix japonica* (JQ340002).

To validate the phylogenetic position of *S. borneensis*, a Bayesian Inference (BI) tree was constructed (Figure 1) using MrBayes version 3.2.6 (Ronquist et al. 2012) with 13 PCGs (10,734 bp) from the mitogenomes of sixteen tetrigid species and one outgroup taxon (*Myrmecophilus manni*), respectively. As shown in Figure 1, T*. tonkinensis* occupied the most basal position, followed by the second branch, *S. borneensis*, which was positioned as a sister group to the remaining Tetrigoidea (posterior probability, PP = 1). These data, suggesting that *T. tonkinensis* is the earliest species within Tetrigoidea, followed by *S. borneensis*, which was consistent with the results of the molecular phylogenetic analyses conducted by earlier study showing that *S. borneensis* is an earlier species within Tetrigoidea (Zhang et al. 2020). Moreover, sixteen tetrigid species were grouped in a clade with strong support (PP = 1), which suggested Tetrigidae is monophyletic.

**Figure 1.**
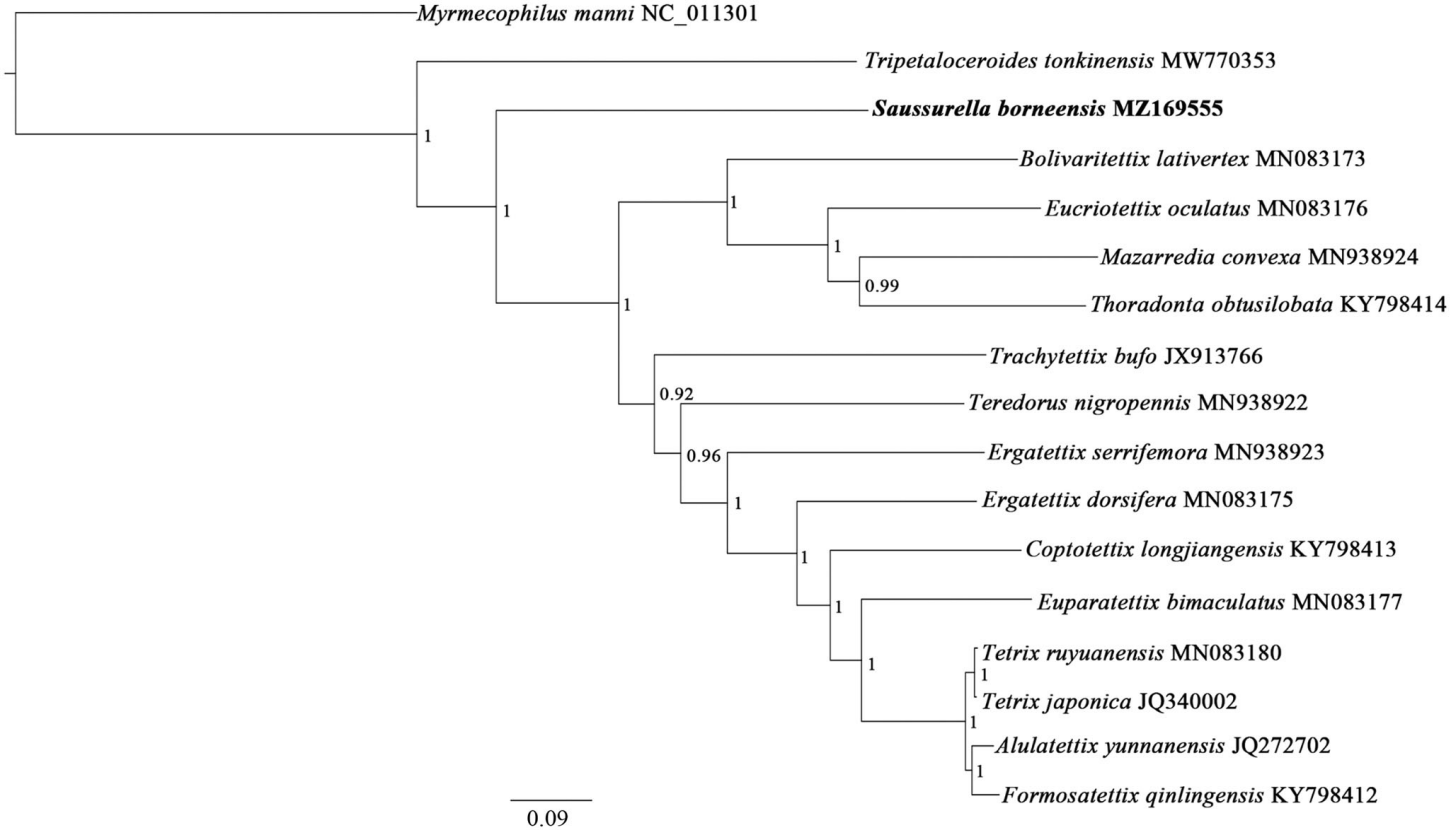
Phylogenetic tree obtained from BI analysis based on 13 concatenated mitochondrial PCGs. Values at nodes indicate BI posterior probabilities (PP).

## Data Availability

The genome sequence data that support the findings of this study are openly available in GenBank of NCBI at [https://www.ncbi.nlm.nih.gov] under the accession no. MZ169555. The associated BioProject, SRA and BioSample numbers are PRJNA743276, SRR15031231, and SAMN20014183, respectively.
